# Involvement of the Soluble Urokinase Receptor in Chondrosarcoma Cell Mobilization

**DOI:** 10.1155/2011/842842

**Published:** 2010-12-30

**Authors:** Katia Bifulco, Immacolata Longanesi-Cattani, Maria Teresa Masucci, Annarosaria De Chiara, Flavio Fazioli, Gioconda Di Carluccio, Giuseppe Pirozzi, Michele Gallo, Antonello La Rocca, Gaetano Apice, Gaetano Rocco, Maria Vincenza Carriero

**Affiliations:** ^1^Department of Experimental Oncology, National Cancer Institute of Naples, Via Mariano Semmola 1, 80131 Naples, Italy; ^2^Department of Human Pathology, National Cancer Institute of Naples, Via Mariano Semmola 1, 80131 Naples, Italy; ^3^Department of Surgical Oncology, National Cancer Institute of Naples, Via Mariano Semmola 1, 80131 Naples, Italy; ^4^Department of Medical Oncology, National Cancer Institute of Naples, Via Mariano Semmola 1, 80131 Naples, Italy

## Abstract

High levels of urokinase receptor (uPAR) in tissue and serum of patients with chondrosarcoma correlate with poor prognosis. First, we analyzed the uPAR levels in tissues and plasma of five patients affected by chondrosarcoma. Interestingly, very high levels of uPAR and its soluble forms (SuPAR) were found on tumor cell surfaces and plasma, respectively, of two patients with lung metastases. Therefore, to investigate the role of SuPAR in chondrosaromas, we generated a primary cell culture from a chondrosarcoma tissue overexpressing uPAR on cell surfaces. We found that chondrosarcoma-like primary culture cells release a large amount of SuPAR in the medium. *In vitro*, SuPAR elicits chondrosarcoma cell migration likely through its uPAR_88-92_ sequence, since the DII_88-183_ or DIIDIIR_88-284_ uPAR domains retain motogen effect whereas DI_1-87_ or DIII_184-284_ domains, both lacking the uPAR_88-92_ sequence, are ineffective. Chondrosarcoma cells cross matrigel in response to SuPAR, and their invasion capability is abrogated by RERF peptide which inhibits uPAR_88-92_ signalling. These findings assign a role to uPAR in mobilizing chondrosarcoma cells and suggest that RERF peptide may be regarded as a prototype to generate new therapeutics for the chondrosarcoma treatment.

## 1. Introduction

Chondrosarcomas are a heterogeneous group of neoplasms having in common the production of cartilage matrix by the tumor cells [1]. Chondrosarcoma accounts for approximately 20% of bone sarcomas with a peak incidence in the fifth to seventh decade of life. Because there are no effective treatments for patients with unresectable or metastatic disease, there is a pressing need to develop new targeted approaches [2]. 

Chondrosarcomas can progress from low grade to high grade, which is reflected by increased cellularity, nuclear atypia, mucomyxoid matrix changes, and increased vascularization [3, 4]. Low-grade chondrosarcomas are locally aggressive but rarely metastasize [5]. By contrast, high-grade chondrosarcomas often metastasize and are lethal in most patients [3]. The molecular mechanisms involved in the progression to high-grade chondrosarcoma are beginning to be unravelled [1]. Furthermore, processes such as tumor cell attachment, migration, and invasion, which are known to be fundamental in carcinoma, have not been similarly explored in chondrosarcoma. 

Proteolytic degradation of the extracellular matrix constituents and modification of cell adhesion properties are required for tumor invasion and metastasis. The urokinase plasminogen activator (uPA) system have an important role in tumorigenesis, extracellular matrix degradation, and migration and invasion of tumor cells [6–10]. Upon binding to uPA, the cell-surface urokinase receptor (uPAR) elicits a variety of cell responses, including cell migration and invasion [11]. Many malignant cultured cell lines and human neoplasms have been characterized by their increased uPAR expression [12, 13], thus suggesting that the inhibition of uPAR activity could be a promising strategy to prevent cancer invasion and metastasis. 

uPAR is a glycosylated glycosylphosphatidylinositol-anchored protein [14] formed by three domains DI, DII, and DIII connected by short linker regions [15]. The Ser^88^-Arg-Ser-Arg-Tyr^92^ (uPAR_88–92_) linker region between DI and DII domains is a protease sensitive region which retains chemotactic activity, even in the form of an isolated SRSRY peptide [16, 17]. The flexibility of this region enables its interaction with a wide variety of ligands [18]. uPAR engagement with uPA favours the exposure of the uPAR_88–92_ sequence which, in turn, promotes cytoskeletal rearrangements and directional cell migration by binding to the G-protein-coupled formyl-peptide receptors (FPRs) [16, 17, 19]. By a drug-design approach based on the conformational analysis of the uPAR_88–92_ sequence, we have recently developed a family of peptides which revealed to be uPAR antagonists in virtue of their ability to prevent uPAR/FPR interaction. Among these, we found that RERF peptide potently inhibits *in vitro* and* in vivo* cell migration and invasion of highly invasive human fibrosarcoma HT1080 cells [20].

In tumor tissues, shedding and/or enzymatic cleavage of uPAR generate truncated forms of uPAR (SuPAR), which are secreted in the extracellular milieu [21–24]. Soluble forms of uPAR have been identified, *in vitro*, in conditioned cell culture medium and, *in vivo*, in serum and urine of patients affected by several type of solid tumors, including sarcomas and chondrosarcamas, and have been significantly associated to a bad prognosis [21–25]. In particular, codetection of a high expression level of uPA, uPAR, and PAI-1 in tumour tissue and of SuPAR in serum of patients affected by soft-tissue sarcoma has been reported to significantly correlate with a shortened overall survival [25]. To gain some insight on the role of soluble forms of uPAR in determining an aggressive phenotype of chondrosarcoma, we have analysed the effects of SuPAR on a primary cell culture derived from an uPAR expressing chondrosarcoma case.

## 2. Materials and Methods

### 2.1. Patients, Tissue and Plasma Collection

Six patients with chondrosarcoma were studied. Surgical removed tumors were routinely processed for the histopathological diagnosis performed according to the WHO classification [26]. A representative sample from each tumor excision was immediately frozen in liquid nitrogen and stored at −80°C until used for immunocytochemistry. A sample from the tumor excision of patient no. 6 was immediately processed for preparation of a primary cell culture. Plasma samples were obtained just before surgery and were stored at −80°C until assayed.

### 2.2. Immunohistochemistry

Frozen sections, corresponding to the largest cross-sectional area of the tumor, were cut, placed on glass slides and subjected to immunostaining with the streptavidin-biotin-peroxidase method, as previously described [27]. Briefly, sections were fixed with 2.5% formaldehyde in phosphate buffered saline (PBS) and incubated overnight at 4°C with diluents (negative control), or 2 *μ*g/mL R4 anti-uPAR monoclonal antibody (mAb), kindly provided by G. Hoyer-Hansen (Finsen Institute, Copenhagen, Denmark). After several washes in PBS, 1 : 200 diluted biotinylated goat anti-mouse immunoglobulins were applied to sections at 23°C for 60 min. Thereafter, sections were incubated with streptavidin-biotinylated horseradish peroxidase complex for additional 30 min and the peroxidase-dependent staining was developed by diaminobenzidine. Slides were counterstained with Mayer's haematoxylin.

### 2.3. Primary Cell Culture

A representative sample from the tumor excision (~1 cm × 1 cm) from patient no. 6 was immediately minced by scalpel under sterile conditions and incubated with 1.0 mg/mL collagenase XI (Sigma) for 3 h at 37°C under gentle agitation, as previously described [28]. Cells, recovered by centrifugation at 1500 rpm, were cultured in 6-well multidish plates in Dulbecco Modified Essential Medium (DMEM) with the addition of 10% foetal bovine serum (FBS), 100 IU/mL penicillin and 50 *μ*g/mL streptomycin. Isolated cell clusters were further amplified in growth medium until an adherent, homogeneous cell population was obtained.

### 2.4. Cell Lines and Conditioned Media

Mouse fibroblast LB6, LB6 cells stably transfected with cDNA encoding human SuPAR (LB6/hSuPAR) [12], and human fibrosarcoma HT1080 cell line were grown in DMEM supplemented with 10%FBS, 100 IU/mL penicillin and 50 *μ*g/mL streptomycin. To prepare conditioned media, LB6, LB6/hSuPAR, or chondrosarcoma cells were grown to 80% confluence on 10 cm Ø plates. Growth medium was removed and cells, after extensive washing with PBS, were incubated in serum-free medium. After 18 h, the medium was recovered, cleared by centrifugation, and analysed for the SuPAR content applying a commercially available enzyme-linked immunosorbent assay kit (ELISA) purchased by R&D System, as previously described [12]. Antigen concentrations were expressed as ng analyte per *μ*g proteins.

### 2.5. Immunofluorescence

Chondrosarcoma cells, plated on glass slides (30%–40% confluence), were fixed and permeabilized with 2.5% formaldeyde-0.2% Triton X-100 in PBS for 10 min at 4°C, then incubated overnight at 4°C with 5 *μ*g/mL anti-vimentin (Dako), anti-cytokeratin (Zymed Laboratories Inc.) or 2 *μ*g/mL R4 anti-uPAR mAbs. A subset of experiments was performed on fixed with 2.5% formaldeyde, nonpermeabilized cells. Immunofluorescence was carried out by incubating slides with 1 : 100 diluted Alexa 488-conjugated F(ab')2 fragment of rabbit anti-mouse IgG (Molecular Probes) for 1h at 22°C. After nuclear staining with 4^'^6-diamidino-2-phenylindole dye (DAPI), cells were analysed by a fluorescence inverted microscope connected to a videocamera (Carl Zeiss), as described [20].

### 2.6. Flow Cytometry

Cells were detached using 200 mg/L EDTA, 500 mg/L trypsin (Cambrex). Nonspecific binding sites, possibly due to any Fc receptor, were blocked by normal rat serum. Cells (0.5 × 10^6^ cells/sample) were incubated with 1 : 40 normal rat serum added to PBS (CTL) or 2 *μ*g/mL R4 anti-uPAR mAb for 30 min at 4°C. After extensive washing with PBS, cells were incubated with Alexa 488-conjugated F(ab')2 fragment of rabbit anti-mouse IgG and finally resuspended in 0.6 mL PBS. Samples were analysed by flow cytometry using a FACS Vantage cell sorter (Becton & Dickinson). All data were analysed using CellQuest software.

### 2.7. Immunoprecipitation

Conditioned medium of LB6, LB6/hSuPAR or chondrosarcoma cells (500 *μ*L/sample) were precleared with 10 *μ*L Protein G-Sepharose (Ge-Healthcare) for 1 hr at 4°C, immunoprecipitated with 100 *μ*L rabbit 399 anti-uPAR conjugated to sepharose beads (0.5 mg IgG per mL of *beads*) diluted 1 : 1 for 18 h at 4°C. Beads were washed and then boiled in SDS-PAGE sample buffer. Proteins were separated by a 12.5% SDS-PAGE followed by Western blotting with 2 *μ*g/mL R4 anti-uPAR mAb.

### 2.8. Cell Migration and Invasion Assays

Cell migration and invasion assays were performed using Boyden chambers and 8 *μ*m pore size polyvinyl-pyrrolidone-free polycarbonate filters (Nucleopore) as previously described [20, 27]. The ability of primary cell culture to migrate or to cross matrigel was assessed between the VI and the IX passage. For cell invasion assays, filters were coated with 50 *μ*g/mL matrigel, a reconstituted basement membrane (BD Biosciences). Cells were preincubated with DMEM, 2 *μ*g/mL normal rabbit serum (NRS), blocking 399 anti-uPAR Ab [29, 30], or anti-uPAR_84–95_ Ab which specifically recognizes the uPAR chemotactic Ser^88^-Arg-Ser-Arg-Tyr^92^ sequence [31] for 1 h at 37°C, prior to seeding in the upper chamber at 3 × 10^4^ cells/well. In a subset of experiments, cells were exposed to 10 nM RERF or ERFR peptides (Primm) which we have previously reported to inhibit uPAR_88–92_-dependent signalling without affecting cell proliferation [20]. The indicated chemoattractants were placed in the bottom well. Recombinant uPAR domains (Calbiochem) were employed at 10 nM concentration. Cells were allowed to migrate or invade matrigel at 37°C in humidified air with 5% CO_2_ for 4 h or 18 h, respectively. At the end of the assay, cells in the upper chamber and on the upper filter surface were removed whereas cells on the lower filter surface were fixed with ethanol and stained with haematoxylin. The number of migrating or invading cells was determined by counting cells in 10 random fields/filter at 200x magnification. HT1080 cells were employed as an internal control. Data were calculated as a percentage of migrated or invading cells in the absence of chemoattractant, considered as 100%.

### 2.9. Statistical Analysis

The data were analysed for significance using Student's *t*-test. Differences were considered statistically significant at a level of *P* < .05. 

## 3. Results and Discussion

### 3.1. uPAR Expression and SuPAR Release in Chondrosarcomas

**Figure 1:**
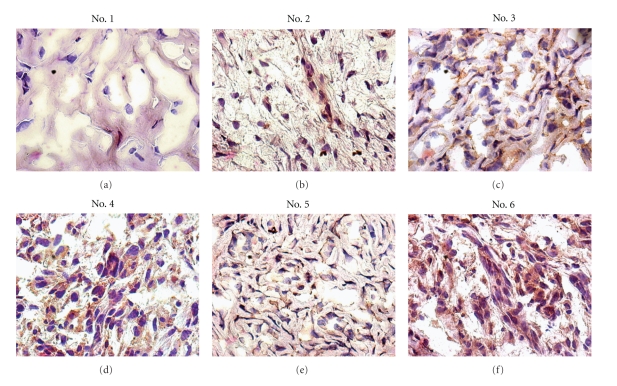
uPAR protein is expressed in chondrosarcoma tissues. Immunohistochemistry was performed on frozen sections with the streptavidin-biotin-peroxidase method using 2 *μ*g/mL R4 anti-uPAR mAb. Sections were counterstained with haematoxylin. Original magnifications: x400.

The age of the patients at diagnosis ranged from 34 to 72 years. Surgical removed tumors were routinely processed for the histopathological diagnosis performed according to the WHO classification [26].  Table 1 reports the pathological findings of 6 primary chondrosarcomas: 5 were primary bone lesions including femur (3) and sternum (2) and 1 was extraskeletal lesion involving pelvis (Table 1). The main *clinical* features at diagnosis are summarized in Table 2. To investigate the molecular mechanisms underlying the activity of uPAR in chondrosarcoma, we first analysed the uPAR expression on chondrosarcoma tissues by immunohistochemistry, using R4 anti-uPAR mAb. The intensity of uPAR staining of tumor cells was graded as faint (grading 1), moderate (grading 2), or intense (grading 3) (Table 2). Except for the benign/low grade lesion which did not show any reactivity to R4 anti-uPAR mAb, all tumors, although at a different extent, exhibit a heterogeneous pattern of staining, mainly localized on tumor plasma cell membranes (Figure 1). Several tumor cells have been reported to shed soluble forms of uPAR [21–25]. Therefore, we performed a quantitative analysis of the of SuPAR content in the plasma of patients using a commercially Elisa Kit. As shown in Table 2, an appreciable amount of SuPAR was detected in all the plasma tested. Interestingly, patients with lung metastases (#3 and #4) exhibited higher levels of SuPAR (Table 2). These data encouraged us to further analyze the role of SuPAR in chondrosaroma invasiveness.

**Figure 2:**
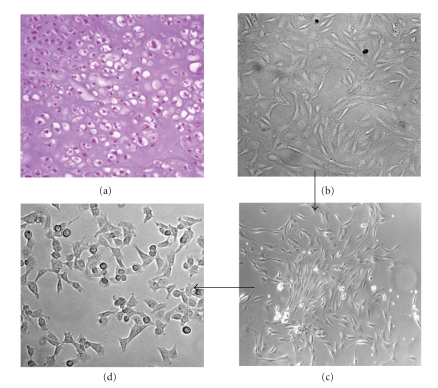
Generation of a primary cell culture from a chondrosarcoma tissue. (a) Haematoxylin/eosin stained section of a human primary grade 2, focally grade 3 chondrosarcoma of sternum (patient no. 6). (b–d) A representative sample from the tumor excision was subjected to enzymatic digestion. The isolated cells, recovered by centrifugation at 1500 rpm, were cultured in DMEM 10% FBS until to the third passage. At this passage, cell population showed an evident cellular heterogeneity (b). Subcloning of the isolated cell clusters (c) and amplification for six further passages resulted in an adherent, homogeneous cell population characterized by small, chondrosarcoma-like cells (d). Images were captured by an inverted microscope connected to a video camera. Original magnification: x200 (a and c), x400 (b and d).

The patient no. 6 underwent a surgery for an extensive sternal mass (Table 1). Preoperative workup showed bilateral nodules suspicious for pulmonary metastases. Following multidisciplinary consultation, it was decided to submit the patient to complete sternal resection and pulmonary metastasectomy. For the reconstruction of the anterior chest wall defect, three cadaveric cryopreserved ribs were used. The resected tumor measured 18 × 15 × 8 cm. Histology of the primary tumor yielded a diagnosis of grade 2, focally grade 3 chondrosarcoma, characterized by frank hypercellularity, with elongated hyperchromatic and sometimes binucleated nuclei (Figure 2(a)). The pulmonary lesions were confirmed to be pulmonary metastases from chondrosarcoma. The patient was followed up to 8 months after primary surgery, when multiple extraskeletal metastases were detected. At this point, he received chemotherapy, but died soon after due to chemotherapy-related complications.

### 3.2. Isolation and Characterization of Chondrosarcoma Cells

**Figure 3:**
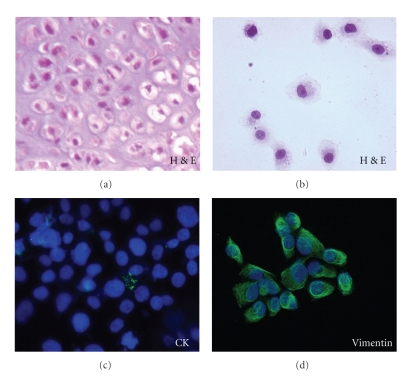
Immunophenotyping of chondrosarcoma cells. (a, b) haematoxilin/eosin stained chondrosarcoma cells resemble in shape and size those of chondrosarcoma tissue section. (c, d) chondrosarcoma cells grown on glass slides to semiconfluence were stained with anti-cytokeratin (CK) or anti-vimentin mAbs and with Alexa 488-conjugated F(ab')2 fragment of rabbit anti-mouse IgG (green). Nuclei were stained blue with DAPI. Original magnification: x400.

As described in the methods, a representative sample from the tumor excision was minced and subjected to enzymatic digestion; cell suspension was recovered and cultured in multidish plates until to the third passage (Figure 2(b)). Subcloning of the isolated cell clusters (Figure 2(c)) and seven further passages resulted in an adherent, homogeneous cell population mainly characterized by small chondrosarcoma-like cells (Figure 2(d)), resembling, in shape and size, those observed on haematoxilin/eosin stained section (Figures 3(a) and 3(b)). Immunocytochemical analysis of cells grown on glass slides revealed the total absence of epithelial (cytokeratin) cell marker whereas a strong staining was observed in the 95% of cells exposed to anti-vimentin mAb (Figures 3(c) and 3(d)).

### 3.3. Identification of Membrane-Anchored uPAR on Chondrosarcoma Cells

**Figure 4:**
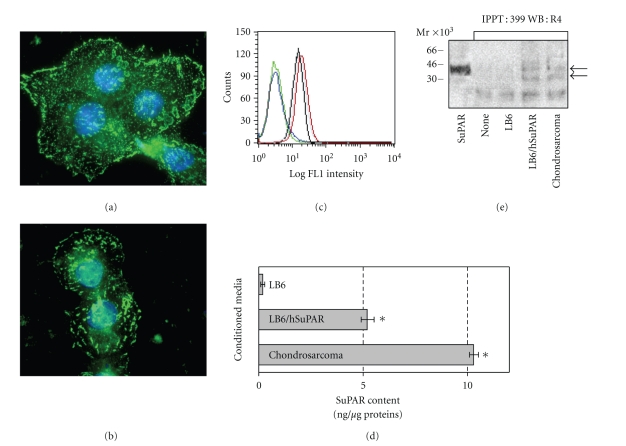
Chondrosarcoma cells express uPAR and release its soluble forms in the medium. Chondrosarcoma cells were fixed with 2.5% formaldeyde (a) or fixed with 2.5% formaldeyde and permeabilized with 0.2% Triton X-100 (b) for 10 min at 4°C, incubated overnight at 4°C with 2 *μ*g/mL R4 anti-uPAR mAb and then exposed to Alexa 488-conjugated F(ab')2 fragment of rabbit anti-mouse IgG (green). Nuclei were stained blue with DAPI. Original magnification: x1000. (c) Flow cytometry analysis of uPAR on chondrosarcoma and HT1080 cell surfaces. HT1080 and chondrosarcoma cells were harvested, incubated with normal rat serum added to PBS (green and blue curves, resp.) or 2 *μ*g/mL R4 anti-uPAR mAb (black and red curves, resp.), stained with Alexa 488-conjugated F(ab')2 rabbit anti-mouse IgG and analyed by FACS. (d) Chondrosarcoma cells release soluble forms of uPAR in the medium. LB6, LB6/hSuPAR, or chondrosarcoma cells were grown to 80% confluence. Growth medium was removed, cells were extensively washed with PBS and then incubated in serum-free medium for 18 h. The medium was recovered, cleared by centrifugation, and analysed for SuPAR content by ELISA. Antigen concentrations were expressed as ng SuPAR per *μ*g proteins. Columns, mean of two independent experiments; bars, ±SD, *P < .001 against the control (conditioned medium of LB6 wild-type cells). (e) 500 *μ*L conditioned media of LB6, LB6/hSuPAR or chondrosarcoma cells were immunoprecipitated with 100 *μ*L rabbit 399 anti-uPAR Ab conjugated to sepharose beads for 18 h at 4°C. The eluted proteins were separated by a 12.5% SDS-PAGE and analysed by Western blot using R4 anti-uPAR mAb. 1 *μ*g purified SuPAR or 10 *μ*L 399 anti-uPAR conjugated to sepharose beads (none) were loaded as a control. Arrows indicate the full-length SuPAR and the DIIDIII fragment of SuPAR.

First, uPAR expression was analysed in chondrosarcoma cells by immunofluorescence and cytofluorimetry. Accordingly to immunohistochemical findings (Figure 1), chondrosarcoma cells express high levels of uPAR mainly localized on plasma cell membranes (Figure 4(a)). In permeabilized cells, a discrete, intracytoplasmic amount of uPAR was also found, thus indicating that chondrosarcoma cells effectively synthesize uPAR (Figure 4(b)). Interestingly, flow cytometry revealed that chondrosarcoma cells express higher levels of uPAR as compared to HT1080 cells (25 and 23,9 mean fluorescence intensity, resp., in the 98% of total cells) (Figure 4(c)).

### 3.4. Chondrosarcoma Cells Shed Soluble Forms of uPAR into the Medium

We investigated whether soluble forms of uPAR are produced and released by chondrosarcoma cells in the culture medium. To this purpose, we took advantage by employing, as a negative and a positive controls, conditioned medium of uPAR lacking wild-type LB6 and transfected LB6/hSuPAR cells, respectively. We measured by Elisa the amount of SuPAR antigen released in the conditioned medium of LB6, LB6/hSuPAR and chondrosarcoma cells. As expected, LB6/hSuPAR cells released a large amount of SuPAR as compared to the wild-type LB6 cells [12]. In keeping with the expression of high levels of uPAR on plasma cell membranes, chondrosarcoma cells released a very large amount of SuPAR in the medium as compared to that produced by LB6/hSuPAR cells (10.3 ng/*μ*g and 5.2 ng/*μ*g of proteins, resp.) (Figure 4(d)). To ascertain the occurrence of cleaved forms of SuPAR in the conditioned medium of chondrosarcoma cells, serum-free medium was subjected to immunoprecipitation with rabbit 399 anti-uPAR Ab which recognizes all soluble forms of SuPAR, followed by Western blotting with R4 anti-uPAR mAb which recognizes full-length DIIDIII and DIII cleaved forms of uPAR [24]. LB6 and LB6/hSuPAR conditioned media were employed as negative and positive control, respectively. According to Sidenius et al. [24], we found in the conditioned medium of both chondrosarcoma and LB6/hSuPAR, but not LB6 cells, a fragment with an approximate 45 kDa molecular weight, comigrating with the purified full-length SuPAR (Figure 4(d)). R4 anti-uPAR mAb specifically recognized in the conditioned medium of LB6/hSuPAR but not LB6 cells an additional fragment having molecular weight of about 35 kDa, compatible with the cleaved DIIDIII fragment of SuPAR. As shown in Figure 4(e), a fragment with an approximate 35 kDa molecular weight, comigrating with the DIIDIII fragment of SuPAR was found in the conditioned medium of chondrosarcoma cells. Although we can not assess whether other fragments of SuPAR do exist in the chondrosarcoma cell medium, this findings indicate that at least two fragments, both containing the chemotactic sequence of uPAR, may influence cell behaviour.

### 3.5. SuPAR Promotes *In Vitro* Migration of Human Chondrosarcoma Cells

Soluble forms of uPAR, including the DIIDIII fragment, have been shown to strongly chemoattract a variety of cell types [16, 17, 20, 32]. We investigated whether chondrosarcoma cells specifically respond to soluble forms of uPAR. In these experiments, we took advantage by employing the uPAR expressing human fibrosarcoma HT1080 cells as an internal control. Cell migration assays were carried in Boyden chambers using conditioned media of wild-type LB6 or LB6/hSuPAR cells. We found that similarly to HT1080, chondrosarcoma cells exhibited a strong ability to migrate toward the LB6/hSuPAR conditioned medium, reaching 363 ± 26% of the random cell migration (Table 3). On the contrary, wild-type LB6 conditioned medium did not exert any effect, indicating that chondrosarcoma cells specifically respond to SuPAR. Interestingly, 10 nM recombinant DII_88–183_ or DIIDIII_88–284_ uPAR domains as well as full length DIDIIDIII_1–284_ triggered an appreciable cell migration (215%  ± 5, 221%  ± 13, and 240%  ± 10, resp.) whereas recombinant DI_1–87_ or DIII_184–284_ uPAR domains, both lacking the uPAR_88–92_ sequence, were ineffective at 10nM (Table 3). These findings suggest that SuPAR is able to mobilize chondrosarcoma cells through its uPAR_88–92_ sequence. To further investigate the role of the uPAR_88–92_ sequence in mobilizing chondrosarcoma cells, a subset of experiments was performed in the presence of 399 anti-uPAR Ab or a polyclonal antibody which specifically recognized the uPAR_84–95_ sequence [30, 31]. As shown in Table 3, in the presence of 399 anti-uPAR or anti- uPAR_84–95_ Abs, both HT1080 and chondrosarcoma cells failed to respond to the conditioned medium of LB6/hSuPAR cells or to recombinant uPAR domains containing the chemotactic sequence. The inhibition was specific, as the presence of nonimmune serum did not abrogate cell motility. All together, these results clearly indicate that soluble forms of uPAR containing the uPAR_88–92_ sequence are able to mobilize chondrosarcoma cells.

### 3.6. uPAR Promotes *In Vitro* Invasion of Human Chondrosarcoma Cells

**Figure 5:**
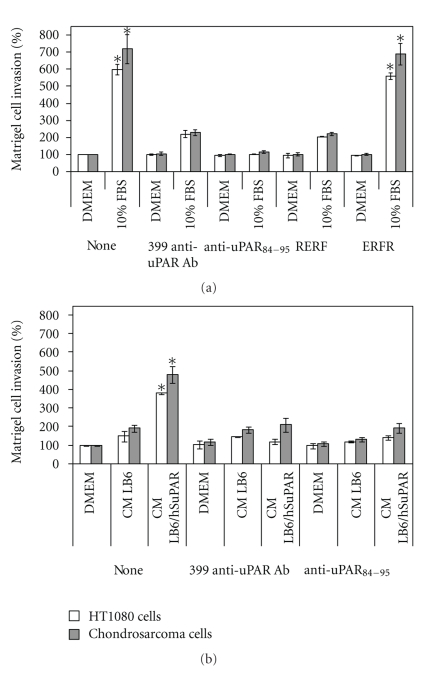
uPAR-dependent matrigel invasion of chondrosarcoma. Cells human fibrosarcoma HT1080 and chondrosarcoma cells were subjected to cell invasion assays. Cells were preincubated with DMEM (none), 2 *μ*g/mL indicated anti-uPAR antibodies, 10 nM RERF peptide or 10 nM ERFR peptide, plated in Boyden chambers and allowed to cross matrigel-coated filters for 18 h at 37°C in humidified air with 5% CO_2_. 10% FBS (a), LB6, or LB6/hSuPAR conditioned media (b) were employed as chemoattractants. In all cases, data are reported as a percentage of invading cells in the absence of chemoattractant, considered as 100% (DMEM), and represent the average of two experiments, performed in duplicate. Columns, mean of two independent experiments; bars, ±SD, *P < .001 against the control (DMEM).

Cell migration is a prerequisite for cancer invasion. Therefore, we performed *in vitro* matrigel invasion assays [33] to quantify, the relative invasive potential of chondrosarcoma cells. In these assays, 10% FBS was employed as a source of chemoattractants and the basal cell invasion, assessed in the absence of any chemoattractant, was taken as 100%. We employed the uPAR expressing human fibrosarcoma HT1080 cells as an internal control. According to their previously reported highly invasive capability [20], HT1080 cells exhibited a strong ability to cross matrigel (597 ± 30% of the basal level). Chondrosarcoma cells exhibited a very high ability to invade matrigel as compared to HT1080 cells, reaching 721 ± 91% of the basal level (Figure 5(a)). Interestingly, cell exposure to blocking 399 anti-uPAR or anti-uPAR_84–95_ Abs strongly reduced cell invasion ability of both HT1080 and chondrosarcoma cells. To further elucidate the role of uPAR in promoting cell invasiveness, a subset of experiments were performed in the presence of RERF peptide which we have previously reported to specifically inhibit uPAR_88–92_-dependent signalling without affecting cell proliferation [20]. We found that cell exposure to 10 nM RERF strongly reduced the ability of both HT1080 and chondrosarcoma cells to cross matrigel whereas the ERFR control peptide failed to exert any inhibitory effect. These findings strongly support the role of uPAR in promoting cell invasion. To assess whether also soluble forms of uPAR may be involved in cell invasion capability, experiments were performed using conditioned medium of LB6/hSuPAR cells as a source of intact and DIIDIII uPAR fragment, or conditioned medium of LB6 cells as a negative control. We found that both HT1080 and chondrosarcoma cells respond to LB6/hSuPAR conditioned medium, the effect being prevented by 399 anti-uPAR as well as by anti-uPAR_84–95_ Abs (Figure 5(b)). 

## 4. Conclusions

uPAR plays a key role in pathological processes sustained by an altered cell migration [11]. High levels of uPAR and SuPAR in tissue and serum of patients with sarcoma, including chondrosarcoma, correlate with a poor prognosis [25]. We generated a primary cell culture derived from a uPAR overexpressing chondrosarcoma tissue. We found that chondrosarcoma-like primary culture cells express high level of uPAR on plasma cell membranes and release a large amount of intact SuPAR as well as DIIDIII uPAR fragment in the medium. Our findings revealed that, *in vitro*, SuPAR: (i) elicits chondrosarcoma cell migration through its uPAR_88–92_ sequence and (ii) promotes chondrosarcoma cell invasion, the effect being reduced to the basal level by anti-uPAR blocking antibody. Taken together, our findings raises the possibility that soluble uPAR released by chondrosarcoma cells in the extracellular matrix may generate a chemotactic gradient which, in turn, stimulates tumor cells to migrate and invade the surrounding tissues. There are few studies investigating the clinical impact of uPAR expression and its correlation to prognosis in chondrosarcoma [25]. Our findings suggest that the determination of plasmatic SuPAR content in patients with chondrosarcoma could be helpful for a prognostic evaluation. Furthermore, there are currently no universally effective therapies for unresectable or metastasized chondrosarcomas [1]. It can be envisaged that the inhibition of uPAR activity could be a promising strategy to prevent chondrosarcoma invasion and metastasis. We found that, *in vitro*, the chondrosarcoma cell invasion ability may be abrogated by RERF peptide which specifically inhibits the uPAR_88–92_-dependent signalling by preventing its interaction with the G-protein coupled formyl-peptide receptor [20]. In conclusion, our data indicate that uPAR is required for the cell migration and invasion machinery of chondrosarcoma cells and suggest that RERF peptide may be regarded as a prototype to generate new therapeutic agents for the treatment of unresectable or metastasized chondrosarcoma.

## Figures and Tables

**Table 1:** Histopathological findings of enrolled chondrosarcoma patients.

Patients	Age (yr)	Gender	Site	Size (cm)	Histology^a^	Grade
1	42	F	Right femur	5.5 × 2 × 2.5	Low-grade chondrosarcoma	
2	34	F	Pelvis	10 × 8 × 8	Mesenchymal chondrosarcoma	G2
3	72	M	Left femur	21 × 12 × 12	Dedifferentiated chondrosarcoma	G3
4	63	F	Right femur	13 × 9 × 28	Dedifferentiated chondrosarcoma	G3
5	69	M	Sternum	10 × 7 × 5	Chondrosarcoma	G2, focallyG3
6	58	M	Sternum	18 × 15 × 8	Chondrosarcoma	G2, focallyG3

^a^Histopathological diagnosis was performed according to the WHO classification. F, female; M, male.

**Table 2:** Clinicopathological parameters and uPAR levels in tumour tissues and in plasma of patients with chondrosarcoma.

Patients	First clinical evaluation	Therapy	Survival from diagnosis (months)	uPAR grading^a^	SuPAR (pg/mL)^b^
1	P	Surgery	6	0	1094
2	P	Surgery	87	1	1.508
3	P + Lung M	Surgery	3	3	3.645
4	P + Lung M	Neoadiuvant chemotherapy and surgery	14	3	5.152
5	P	Surgery	9	2	1.556
6	P + Lung M	Surgery and chemotherapy	8	3	ND

P, primary tumor; M, metastasis; ^a^Immunohistochemical staining of tumor frozen sections with R4 anti-uPAR mAb was graded as absent (grading 0), faint (grading 1), moderate (grading 2), or intense (grading 3). ^b^Determination of plasmatic SuPAR content by Elisa, expressed as pg SuPAR/mL plasma.

**Table 3:** SuPAR-dependent migration of chondrosarcoma cells.

Supplements	Effector	Cell migration (%)	
		HT1080 cells	Chondrosarcoma cells
DMEM	DMEM	100	100
	CM LB6	110 ± 5	112 ± 8
	CM LB6/SuPAR	401 ± 12**	363 ± 16**
	10nM DI_1–87_	115 ± 6	105 ± 8
	10nM DII_88–183_	265 ± 9**	215 ± 5**
	10nM DIII_184–284_	98 ± 9*	100 ± 6
	10nM DIIDIII_88–284_	259 ± 11**	221 ± 13*
	10nM DIDIIDIII_1–284_	270 ± 15*	240 ± 10**

NRS	DMEM	100 ± 3	100 ± 2
	CM LB6/SuPAR	398 ± 9**	351 ± 6**
	10nM DII_88–183_	271 ± 5**	225 ± 11**
	10nM DIIDIII_88–284_	262 ± 9**	200 ± 8**
	10nM DIDIIDIII_1–284_	250 ± 9*	236 ± 13*

399 Anti-uPAR Ab	DMEM	100 ± 7	100 ± 5
	CM LB6/SuPAR	145 ± 12	133 ± 10
	10nM DII_88–183_	115 ± 6	124 ± 9
	10nM DIIDIII_88–284_	106 ± 11	99 ± 13
	10nM DIDIIDIII_1–284_	110 ± 17	102 ± 14

Anti-uPAR_84–95_ Ab	DMEM	100 ± 6	100 ± 5
	CM LB6/SuPAR	124 ± 9	130 ± 6
	10nM DII_88–183_	104 ± 13	101 ± 7
	10nM DIIDIII_88–284_	102 ± 9	119 ± 15
	10nM DIDIIDIII_1–284_	107 ± 11	110 ± 13

Cells incubated with diluents (DMEM) or 5 *μ*g/mL the indicated antibody for 1 h at 37°C were seeded in Boyden chambers for cell migration assays as described in the Material and Method section, in the presence or absence of the indicated effectors. Conditioned medium (CM) of LB6/hSuPAR cells was used as a source of SuPAR. For quantitative analysis of cell migration, the basal value (DMEM) was taken as 100% and all values were reported relative to that. Data are the means ± SD of two independent experiments, performed in duplicate. Statistical significance with *P* values was calculated against the control DMEM. *Statistical significance with *P* < .05. **Statistical significance with *P* < .001.

## References

[1] Bovée JVMG, Hogendoorn PCW, Wunder JS, Alman BA (2010). Cartilage tumours and bone development: molecular pathology and possible therapeutic targets. *Nature Reviews Cancer*.

[2] Giuffrida AY, Burgueno JE, Koniaris LG, Gutierrez JC, Duncan R, Scully SP (2009). Chondrosarcoma in the United States (1973 to 2003): an analysis of 2890 cases from the SEER database. *Journal of Bone and Joint Surgery - Series A*.

[3] Fiorenza F, Abudu A, Grimer RJ (2002). Risk factors for survival and local control in chondrosarcoma of bone. *Journal of Bone and Joint Surgery - Series B*.

[4] Ayala G, Liu C, Nicosia R, Horowitz S, Lackman R (2000). Microvasculature and VEGF expression in cartilaginous tumors. *Human Pathology*.

[5] Gelderblom H, Hogendoorn PCW, Dijkstra SD (2008). The clinical approach towards chondrosarcoma. *Oncologist*.

[6] Duffy MJ, Duggan C (2004). The urokinase plasminogen activator system: a rich source of tumour markers for the individualised management of patients with cancer. *Clinical Biochemistry*.

[7] Mondino A, Blasi F (2004). uPA and uPAR in fibrinolysis, immunity and pathology. *Trends in Immunology*.

[8] Pillay V, Dass CR, Choong PFM (2007). The urokinase plasminogen activator receptor as a gene therapy target for cancer. *Trends in Biotechnology*.

[9] Binder BR, Mihaly J, Prager GW (2007). uPAR-uPA-PAI-1 interactions and signaling: a vascular biologist’s view. *Thrombosis and Haemostasis*.

[10] Pepper MS (2001). Role of the matrix metalloproteinase and plasminogen activator-plasmin systems in angiogenesis. *Arteriosclerosis, Thrombosis, and Vascular Biology*.

[11] Smith HW, Marshall CJ (2010). Regulation of cell signalling by uPAR. *Nature Reviews Molecular Cell Biology*.

[12] Carriero MV, Franco P, Del Vecchio S (1994). Tissue distribution of soluble and receptor-bound urokinase in human breast cancer using a panel of monoclonal antibodies. *Cancer Research*.

[13] Danø K, Behrendt N, Høyer-Hansen G (2005). Plasminogen activation and cancer. *Thrombosis and Haemostasis*.

[14] Rasch MG, Lund IK, Almasi CE, Hoyer-Hansen G (2008). Intact and cleaved uPAR forms: diagnostic and prognostic value in cancer. *Frontiers in Bioscience*.

[15] Ploug M, Ellis V (1994). Structure-function relationships in the receptor for urokinase-type plasminogen activator: comparison to other members of the Ly-6 family and snake venom *α*-neurotoxins. *FEBS Letters*.

[16] Resnati M, Pallavicini I, Wang JM (2002). The fibrinolytic receptor for urokinase activates the G protein-coupled chemotactic receptor FPRL1/LXA4R. *Proceedings of the National Academy of Sciences of the United States of America*.

[17] Gargiulo L, Longanesi-Cattani I, Bifulco K (2005). Cross-talk between fMLP and vitronectin receptors triggered by urokinase receptor-derived SRSRY peptide. *Journal of Biological Chemistry*.

[18] Barinka C, Parry G, Callahan J (2006). Structural basis of interaction between urokinase-type plasminogen activator and its receptor. *Journal of Molecular Biology*.

[19] Selleri C, Montuori N, Ricci P (2006). *In vivo* activity of the cleaved form of soluble urokinase receptor: a new hematopoietic stem/progenitor cell mobilizer. *Cancer Research*.

[20] Carriero MV, Longanesi-Cattani I, Bifulco K (2009). Structure-based design of an urokinase-type plasminogen activator receptor-derived peptide inhibiting cell migration and lung metastasis. *Molecular Cancer Therapeutics*.

[21] Sidenius N, Blasi F (2003). The urokinase plasminogen activator system in cancer: recent advances and implication for prognosis and therapy. *Cancer and Metastasis Reviews*.

[22] Henić E, Borgfeldt C, Christensen IBJ, Casslén B, Høyer-Hansen G (2008). Cleaved forms of the urokinase plasminogen activator receptor in plasma have diagnostic potential and predict postoperative survival in patients with ovarian cancer. *Clinical Cancer Research*.

[23] Shariat SF, Roehrborn CG, McConnell JD (2007). Association of the circulating levels of the urokinase system of plasminogen activation with the presence of prostate cancer and invasion, progression, and metastasis. *Journal of Clinical Oncology*.

[24] Sidenius N, Sier CFM, Blasi F (2000). Shedding and cleavage of the urokinase receptor (uPAR): identification and characterisation of uPAR fragments in vitro and in vivo. *FEBS Letters*.

[25] Taubert H, Würl P, Greither T (2010). Co-detection of members of the urokinase plasminogen activator system in tumour tissue and serum correlates with a poor prognosis for soft-tissue sarcoma patients. *British Journal of Cancer*.

[26] Antonescu CR, Ladanyi M (2002). *Pathology and Genetics of Tumors of Soft and Bone*.

[27] Silvestri I, Cattani IL, Franco P (2002). Engaged urokinase receptors enhance tumor breast cell migration and invasion by upregulating *α*v*β*5 vitronectin receptor cell surface expression. *International Journal of Cancer*.

[28] Bifulco K, De Chiara A, Fazioli F (2008). Cell invasiveness in sarcomas: a possibly useful clinical correlation. *Tumori*.

[29] Carriero MV, Del Vecchio S, Capozzoli M (1999). Urokinase receptor interacts with *α*(v)*β* vitronectin receptor, promoting urokinase-dependent cell migration in breast cancer. *Cancer Research*.

[30] Bifulco K, Longanesi-Cattani I, Gala M (2010). The soluble form of urokinase receptor promotes angiogenesis through its SER(88) -ARG-SER-ARG-TYR(92) chemotactic sequence. *Journal of Thrombosis and Haemostasis*.

[31] Montuori N, Bifulco K, Carriero MV The cross-talk between the urokinase receptor and fMLP receptors regulates the activity of the CXCR4 chemokine receptor.

[32] Selleri C, Montuori N, Ricci P (2005). Involvement of the urokinase-type plasminogen activator receptor in hematopoietic stem cell mobilization. *Blood*.

[33] Albini A, Iwamoto Y, Kleinman HK (1987). A rapid in vitro assay for quantitating the invasive potential of tumor cells. *Cancer Research*.

